# Risk Factors for Fever After Esophageal Endoscopic Submucosal Dissection and Its Derived Technique

**DOI:** 10.3389/fmed.2022.713211

**Published:** 2022-02-22

**Authors:** Foqiang Liao, Zhenhua Zhu, Yongkang Lai, Xiaolin Pan, Shunhua Long, Xiaojiang Zhou, Guohua Li, Yin Zhu, Youxiang Chen, Xu Shu

**Affiliations:** ^1^Department of Gastroenterology, The First Affiliated Hospital of Nanchang University, Nanchang, China; ^2^Human Genetic Resources Center, The First Affiliated Hospital of Nanchang University, Nanchang, China

**Keywords:** endoscopic submucosal dissection, endoscopic submucosal tunnel dissection, esophageal lesions, fever, risk factors

## Abstract

**Background:**

Fever is one of the postoperative adverse events of endoscopic submucosal dissection and its derived technique, but the probability and risk factors of postoperative fever are still unclear. The aim of the current study was to investigate the incidence and risk factors of postoperative fever after esophageal lesion removal.

**Methods:**

We conducted a retrospective study of 446 patients who underwent esophageal endoscopic submucosal dissection and its derived technique between January 2014 and January 2020. Cases included in this study were divided into fever and non-fever groups.

**Results:**

Postoperative fever developed in 135 patients (30.3%). The median (range) highest fever temperature was 38 (37.8–38.4)°C, the median (range) duration of fever was 1 (1–2) day, and 127 (94.1%) patients developed fever within 24 h after operation. Through logistic regression analysis, factors associated with postoperative fever were age (OR: 1.740, 95% CI: 1.005–3.013, *p* = 0.048), lesion size (OR: 2.007, 95% CI: 1.198–3.362, *p* = 0.008), operation time (OR: 3.007, 95% CI: 1.756–5.147, *p* < 0.001) and nasogastric tube placement (OR: 1.881, 95% CI: 1.165–3.037, *p* = 0.010), while prophylactic antibiotics (OR: 0.181, 95% CI: 0.082–0.401, *p* < 0.001) were negatively associated with fever.

**Conclusions:**

Age ≥52 years old, lesion size ≥19 mm, operation time ≥37 min, and nasogastric tube placement are risk factors for postoperative fever after esophageal endoscopic submucosal dissection and its derived technique, prophylactic antibiotic use after operation may help reduce fever rate. Attention should be paid to such patients to minimize the risk of postoperative fever.

## Introduction

Endoscopic submucosal dissection (ESD) and its derived technique is an advanced endoscopic method, which is increasingly used in esophageal lesions and early esophageal cancer (1–3). However, esophageal ESD is more difficult than other procedures because the anatomy of the esophagus wall is markedly different from the rest of the gastrointestinal tract (4). For relatively large lesions, mucosal dissection affects the surgical field of vision and operating space, further increasing the difficulty of surgery (5). Therefore, endoscopic submucosal tunnel dissection (ESTD) is one of the derivations of standard ESD that is intended to overcome these problems (6). However, some adverse events (such as bleeding, perforation and stenosis) still occur frequently after ESD/ESTD operation (7, 8), and a large number of clinical studies have been conducted on these adverse events and their risk factors (9–11).

At the same time, fever is also a clinically observed postoperative adverse event, and several studies have discussed possible risk factors for fever after ESD in the stomach and colon (12, 13). Nakanishi et al. (12) suggested that age > 68 years, a resection diameter of > 35.0 mm, and increased serum CRP levels at postoperative day 1(POD 1) were independent risk factors for pyrexia after gastric ESD. In colon ESD, logistic regression analysis also revealed that age and lesion size were closely associated with post-ESD fever, but the possibility of bacteremia causing fever is very low (13). However, the sample size of these studies was not large, and the risk factors that may cause postoperative fever have not been fully explored. Moreover, current research on postoperative fever and its risk factors after esophageal ESD and its derived technique is rare. Therefore, we designed this study to explore the incidence and related risk factors of fever after endoscopic submucosal dissection and its derived technique for esophageal lesions to systematically analyze the risk factors that may lead to fever before, during and after operation, so as to reduce the postoperative fever rate by optimizing preoperative risk assessment and strengthening intraoperative and postoperative management.

## Materials and Methods

### Patients and Data Collection

We retrospectively analyzed medical records and endoscopy electronic databases of the First Affiliated Hospital of Nanchang University, and we collected the following demographic data and clinicopathological features: (1) Pre-operative metrics: sex, age, underlying diseases (diabetes, hypertension and history of abdominal surgery), routine blood work, CRP, procalcitonin; (2) intra-operative metrics: lesion location, lesion size, invasion depth, operation method, en-bloc resection, duration of operation, intraoperative bleeding, perforation and management; (3) post-operative metrics: postoperative pathology, prophylactic antibiotics, adverse events (bleeding, perforation, chest pain, nausea and vomiting), body temperature, duration of fever, inflammatory biomarkers (routine blood work, CRP, procalcitonin, blood culture etc.), cooling measures and duration of hospitalization. Between January 2014 and January 2020, 467 patients who underwent ESD/ESTD due to esophageal lesions were included. Cases were excluded for the following reasons: (1) underwent ESD on other body parts or multiple esophageal ESD procedures in the meantime; (2) immunodeficiency status; (3) serious cardiovascular, pulmonary, or hepatorenal diseases; (4) transfer to the surgical department during the ESD/ESTD procedure; (5) fever (temperature >37.5°C) before the procedure; or (6) patients with incomplete demographic data. All included cases were recorded in the Human Genetic Resources Center of the First Affiliated Hospital of Nanchang University. An ethics committee approved the study, and informed consent was obtained from every patient.

### Operative Procedures

Preoperative evaluation (routine blood work, blood biochemistry, coagulation function, ECG, etc.) was completed for all patients after admission. Magnified chromoendoscopy, endoscopic ultrasound, computerized tomography and biopsy were used to estimate characteristics of the lesions, such as invasion depth and possible histologic type. The patient began a fluid diet 1 day before operation and was forbidden to eat for 8 h before operation.

**Figure 1:**
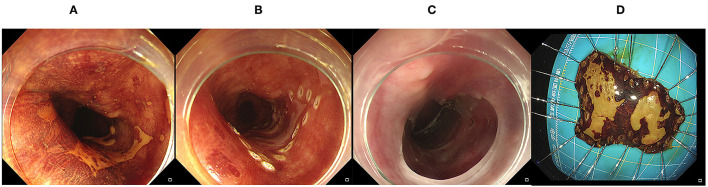
Endoscopic submucosal dissection. **(A)** A Lesion was identified by magnified chromoendoscopy; **(B)** Marking the edge of the target lesion; **(C,D)** After submucosal dissection, the lesion was completely removed.

**Figure 2:**
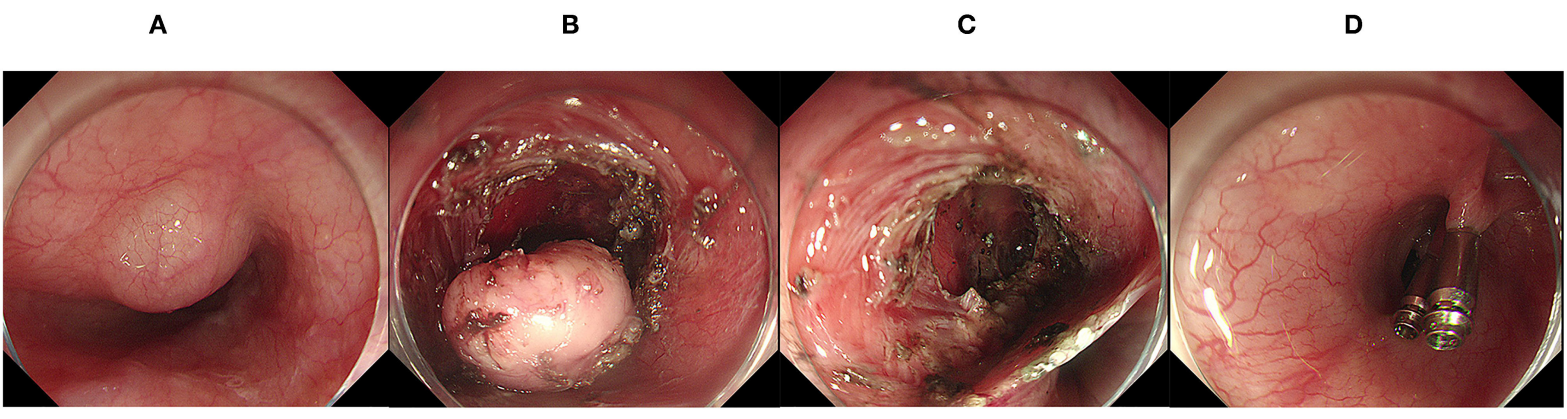
Endoscopic submucosal tunnel dissection. **(A)** A mucosal lesion of the esophagus was found under endoscopy; **(B)** The lesion was exposed after the establishment of the submucosal tunnel; **(C)** The wound surface after endoscopic submucosal tunnel dissection; **(D)** Clips was used to close the defect.

All operative procedures were performed by expert endoscopists with more than 10 years of experience. Patients were sedated after general anesthesia, and ESD was performed in all patients using an electric endoscope (GIF-Q260J; Olympus Optical Co, Ltd, Tokyo, Japan). The operation procedures are as follows (14–16): (1) identification and marking of the target lesion; (2) injection of a lifting solution (0.9% normal saline, glycerol fructose or epinephrine solution) around the perimeter of the lesion; (3) incision of the mucosa along the periphery of the marker dots with ESD knives (hook knife, dual knife, IT knife, etc.); and (4) circumferential mucosal incision and submucosal dissection (Figure 1). The ESTD procedure was performed as follows (17): (1) identification and marking of the target lesion at 3–5 cm with a lifting solution; (2) incision at the marker dots and establishment of the submucosal tunnel by gradually separating the submucosal layer and muscularis to the distal end; (3) resection of the lesion was with a scalpel and removal; and (4) finally, closure of the defect with clips after mucotomy (Figure 2). Hot biopsy forceps or hemostatic forceps were usually used to control bleeding during the procedure. For patients with a large resection area or deep wound, nasogastric tube placement was performed for gastrointestinal decompression, and the color of drainage tube was observed. Experts will decide whether to use prophylactic antibiotics according to the intraoperative situation (included lesion size, operation time and intraoperative perforation), which are high risk factors for postoperative infection.

### Definitions

Operation time was defined as the period from the start of the circumferential mucosal incision to the removal of the tumor (18). Intraoperative bleeding was defined as any exudation or active bleeding that occurred during the procedure. Intraoperative perforation was defined as a visible hole in the esophageal wall, exposing the mediastinal space during the endoscopic procedure (19). En-bloc resection was defined as whole resection of the tumor into a non-fragmenting piece (20, 21). Lesion size was the maximum diameter measured by postoperative pathological specimens. Malignant lesions included esophageal squamous cell carcinoma and adenocarcinoma, while others were classified as benign. Lesions invading the muscularis were defined as having muscle fibers of the muscularis visible by endoscopy. Fever was defined based on a maximum body temperature >37.5°C within 3 days after operation, regardless of the duration of the febrile period. Prophylactic antibiotics refer to the immediate use of antibiotics after ESD/ESTD, but the use of antibiotics after fever was not counted. Postoperative bleeding was defined as hematochezia or melena requiring an endoscopic hemostatic procedure anytime between 0 and 7 days after ESD/ESTD. Postoperative perforation was defined as radiographic evidence of free air after the procedure.

### Statistical Analysis

SPSS software version 25.0 (IBM; Chicago, IL, USA) was used for statistical analysis. Patients were divided into fever and non-fever groups according to the maximum body temperature after operation. Categorical data were compared using Fisher's exact test or the χ^2^ test. Student's *t*-test or the Mann-Whitney *U*-test was used for analysis of quantitative data. Receiver operating characteristic (ROC) curve analysis and Youden index were performed to determine the optimal cut-off values of quantitative data, such as age, tumor size, and procedure time (Supplementary Figure 1). In the univariate analysis to determine independent risk factors for fever, the risk factors were estimated by calculating the odds ratios (ORs) and the 95% confidence intervals (CIs). Variables with *p* < 0.20 in the univariate analysis were included in the multiple logistic regression analysis. *P* < 0.05 were considered to indicate a statistically significant difference between 2 groups.

## Results

### Characteristics of Patients and Lesions

**Figure 3:**
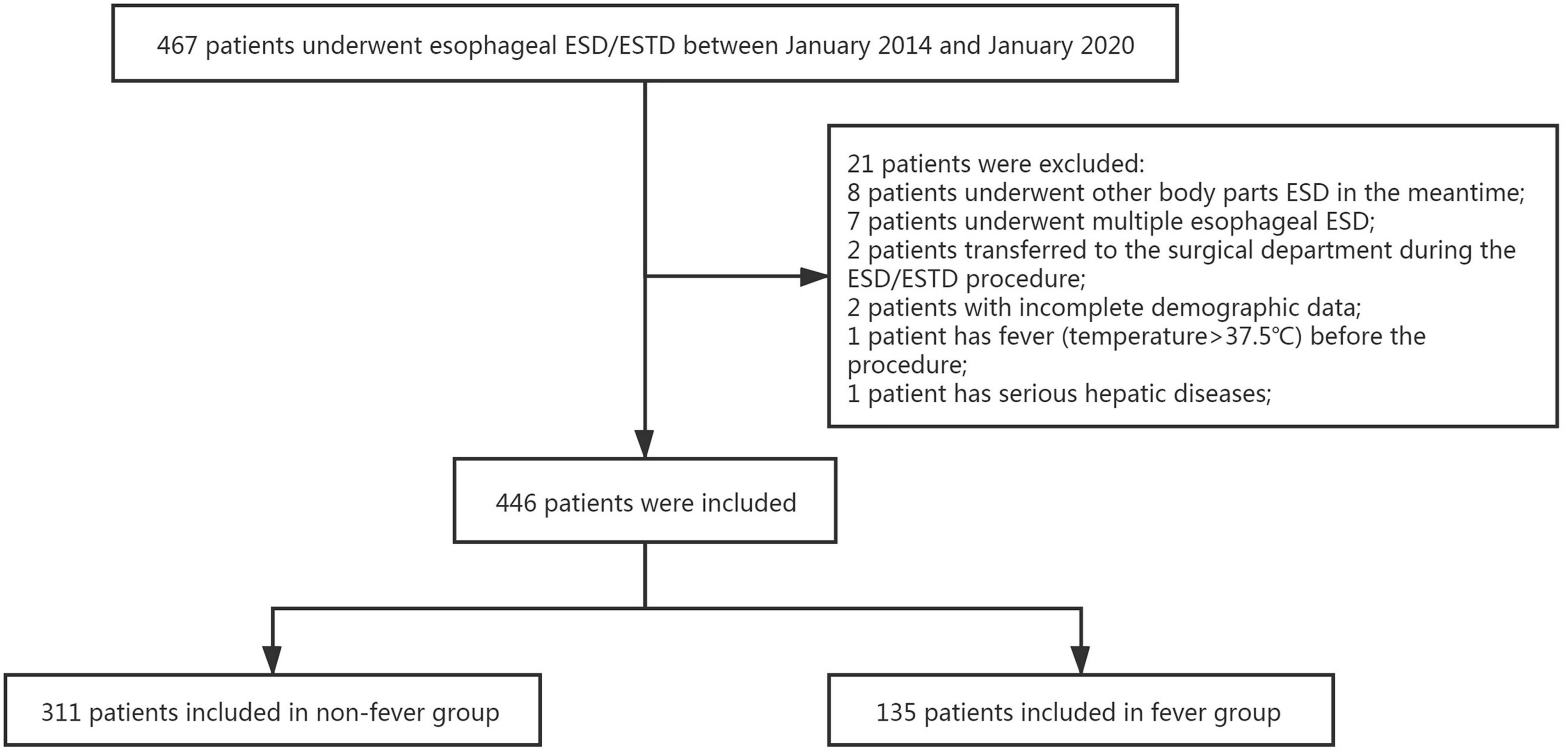
The flowchart of patients with postoperative fever after ESD/ESTD.

We obtained data from 467 patients through the computerized patient record system and endoscopy electronic databases in the First Affiliated Hospital of Nanchang University. Figure 3 shows a total of 446 patients were included in this study. The baseline characteristics of these patients who underwent esophageal ESD/ESTD are presented in Table 1. The median (range) age of study subjects was 57 (47–64) years, and 64.6% (288/446) were male. One hundred twenty-four (27.7%) patients had underlying diseases: 9 (2.0%) patients had diabetes, 66 (14.7%) patients had hypertension, and 49 (11.0%) patients had a history of abdominal surgery. Twelve (2.7%) lesions were located in the upper 1/3 of the esophagus, 348 (78.0%) lesions were located in the middle 1/3 of esophagus and 86 (19.3%) lesions were located in the lower 1/3 of the esophagus. Median (range) lesion size was 15 (10–25) mm. Most lesion were leiomyomas (49.7%), 20.4% (91/446) of lesions were squamous cell carcinomas and 59 (13.2) lesions were dysplasia. Lesions were removed by ESD in 328 (73.5%) patients and ESTD in only 118 (26.5%). Median (range) operation time was 35 (23–58) min, and the en-bloc resection rate was 89.4% (399/446). One hundred seventy-six (39.4%) patients had bleeding during the procedure, and most patients were successfully hemostatic with hot biopsy forceps or hemostatic forceps. Esophageal perforation occurred in 44 (9.8%) patients, and most perforations were closed with clip. Seventy-two (16.1%) patients received prophylactic antibiotics after operation.

**Table 1:** Characteristics of patients and lesions.

**Characteristic**		***N* = 446**
Age [years, median (IQR)]		57 (47–64)
Sex [n(%)]		
	Female	158 (35.4)
	Male	288 (64.6)
Underlying diseases [n(%)]		
	Diabetes	9 (2.0)
	Hypertension	66 (14.7)
	History of abdominal surgery	49 (11.0)
Lesion location within esophagus [n(%)]		
	Upper 1/3	12 (2.7)
	Middle 1/3	348 (78.0)
	Lower 1/3	86 (19.3)
Lesion size [mm, median (IQR)]		15 (10–25)
Pathology [n(%)]		
	Leiomyoma	222 (49.7)
	Squamous carcinoma	91 (20.4)
	Dysplasia	59 (13.2)
	Inflammation	13 (2.9)
	Granular cell tumor	8 (1.8)
	Stromal tumor	6 (1.3)
	Spindle cell tumor	7 (1.6)
	Cyst	7 (1.6)
	Adenocarcinoma	3 (0.7)
	Polyp	3 (0.7)
	Lipoma	3 (0.7)
	Neurofibroma	3 (0.7)
	Others	21 (4.7)
Operation method [n(%)]		
	ESD	328 (73.5)
	ESTD	118 (26.5)
Intraoperative bleeding [n(%)]		176 (39.4)
Perforation [n(%)]		44 (9.8)
Operation time [min, median (IQR)]		35 (23–58)
En-bloc resection [n(%)]		399 (89.4)
Prophylactic antibiotics [n(%)]		72 (16.1)
	Cephalosporin	66 (91.7)
	Others	6 (8.3)
Fever patient [n(%)]		135 (30.3)
	Postoperative Day 1	127 (94.1)
	Postoperative Day 2	5 (3.7)
	Postoperative Day 3	3 (2.2)
Duration of fever [days, median (IQR)]		1 (1–2)
Maximum temperature post operation [°C, median (IQR)]		38 (37.8–38.4)
Treatment measures [n(%)]		
Observation or physical cooling		87 (64.4)
Antibiotics use		26 (19.3)
Antibiotics use + physical cooling		22 (16.3)
Nasogastric tube placement [n(%)]		205 (45.9)
Duration of nasogastric tube placement [days, median (IQR)]		3 (2–4)
Hospitalization [days, median (IQR)]		8 (6–10)
Postoperative adverse events [n(%)]		60 (13.5)
	Bleeding	3 (0.6)
	Perforation	1 (0.2)
	Chest pain	45 (10.1)
	Nausea and vomiting	11 (2.4)
Secondary endoscopic therapy [n(%)]		6 (1.3)
Death rate [n(%)]		0

Postoperative fever rate was 30.3% (135/446), the median (range) of maximum body temperature was 38 (37.8–38.4)°C, the median (range) duration of fever was 1 (1–2) days, all patients developed fever within 3 days of operation, 127 (94.1%) patients developed fever on postoperative day 1, 5 (3.7%) patients developed fever on postoperative day 2, and 3 (2.2%) developed fever on postoperative day 3. All patients with fever recovered after conservative treatment (observation or physical cooling) or antibiotic treatment. Eighty-seven patients recovered to normal body temperature through clinical observation or physical cooling alone, 26 patients were treated with antibiotics, and 22 patients were treated with combined physical cooling and antibiotics. There were no serious complications associated with fever. Two hundred and five (45.9%) patients underwent nasogastric tube placement after operation, and the median (range) period of nasogastric tube placement was 3 (2–4) days. The median (range) duration of hospitalization was 8 (6–10) days, and 60 (13.5%) patients experienced adverse events post-ESD/ESTD, among them, bleeding in 3 (0.6%) patients, perforation in 1 (0.2%) patient, chest pain in 45 (10.1%) patients and nausea and vomiting in 11 (2.4%) patients. Symptoms improved after anti-infection and acid inhibition treatment. Finally, 6 (1.3%) patients received secondary endoscopic surgery due to postoperative pathology, indicating positive surgical margins.

### Comparison Between Non-fever and Fever Groups

Of the 446 patients divided into 2 groups according to whether they developed fever post-ESD/ESTD, 311 (69.7%) patients were included in the non-fever group, and 135 (30.3%) patients were included in fever group. There were several significant differences between patients with vs. without fever post-ESD/ESTD (Table 2). The median age of patients in the fever group was significantly higher compared to the non-fever group (*p* < 0.05). Similarly, median operation time in the fever group was significantly longer compared to the non-fever group (*p* < 0.05). Fever occurred in 46.8% of patients with malignant pathology and 26.9% of patients with benign pathology, indicating a statistical difference between the two groups (*p* < 0.05). Lesions in the fever group were mostly confined to the mucosa/submucosa layer, but lesions in the non-fever group had invaded the muscularis (*p* < 0.05). Median (range) lesion size in the fever group was 22.5 (14.75–35.25) mm but only 15 (10–20) mm in the non-fever group (*p* < 0.05). Significant differences were observed in prophylactic antibiotic use between the fever and non-fever groups (*p* < 0.05). Compared to the non-fever group, the fever group exhibited a significant increase in intraoperative bleeding rate (*p* < 0.05), whereas there was no significant difference in intraoperative perforation rate between the two groups (*p* = 0.814). The rate of nasogastric tube placement was 60.7% (82/135) in the fever group, which was significantly higher than the 39.9% (124/311) observed in the non-fever group. Median hospital stay was longer in patients with fever (9 days; range 7–11 days) compared to those without fever (7 days; range 6–9 days; *P* < 0.001). Finally, there was no significant difference in sex, comorbidities (diabetes, hypertension and history of abdominal surgery), lesion location, operation method or en-bloc resection rate between the two groups. Receiver operating characteristic (ROC) curves were plotted to determine the optimal cut-off values for age, lesion size and operation time to predict fever (Supplementary Figure 1). The cutoff points of the age, lesion size and operation time were 52 years, 19 mm and 37 min, respectively. Operation time ≥37 min was better at distinguishing high-risk patients with fever, with a sensitivity of 70.4%, specificity of 65.3% and the AUC of ROC curve was 0.703. The sensitivity, specificity and AUC for predicting fever were 77%, 42.1% and 0.618 for age ≥52 years. And the sensitivity, specificity and AUC for predicting fever were 64.2, 64.7, and 0.695% for lesion size ≥19 mm (Table 3).

**Table 2:** Comparison between non-fever and fever groups.

	**Non-fever group** **(***n*** = 311)**	**Fever group** **(***n*** = 135)**	**P-Value**
Age [years, median (IQR)]	54 (46–63)	61 (52–65)	<0.001
Sex [n(%)]			0.368
Female	106 (34.1)	52 (38.5)	
Male	205 (65.9)	83 (61.5)	
Underlying diseases [n(%)]			
Diabetes	5 (1.6)	4 (3.0)	0.275
Hypertension	44 (14.1)	22 (16.3)	0.557
History of abdominal surgery	35 (11.3)	14 (10.4)	0.784
Lesion location within esophagus [n(%)]			0.418
Upper 1/3	8 (2.6)	4 (3.0)	
Middle 1/3	238 (76.5)	110 (81.5)	
Lower 1/3	65 (20.9)	21 (15.6)	
Lesion size [mm, median (IQR)]	15 (10–20)	22.5 (14.75–35.25)	<0.001
Pathology [n(%)]			<0.001
Benign	261 (74.1)	91 (25.9)	
Malignant	50 (53.2)	44 (46.8)	
Invasion depth [n(%)]			<0.001
Mucosa/Submucosa	122 (39.2)	80 (59.3)	
Muscularis	189 (60.8)	55 (40.7)	
Operation method [n(%)]			0.071
ESD	221 (71.1)	107 (79.3)	
ESTD	90 (28.9)	28 (20.7)	
Prophylactic antibiotics [n(%)]	63 (20.3)	9 (6.7)	<0.001
Intraoperative bleeding [n(%)]	103 (33.1)	73 (54.1)	<0.001
Perforation [n(%)]	30 (9.6)	14 (10.4)	0.814
Operation time [min, median (IQR)]	30 (21–48)	53 (31.75–75.25)	<0.001
En-bloc resection [n(%)]	278 (89.4)	121 (89.6)	0.939
Nasogastric tube placement [n(%)]	124 (39.9)	82 (60.7)	<0.001
Hospitalization [days, median (IQR)]	7 (6–9)	9 (7–11)	<0.001

**Table 3:** Fever at optimal score thresholds of age, lesion size and operation time.

	**Sensitivity**	**Specificity**	**Youden index**	**AUC**	**95% CI**
Age (≥52 years)	0.770	0.421	0.191	0.618	0.563–0.674
Lesion size (≥19 mm)	0.642	0.647	0.289	0.695	0.641–0.748
Operation time (≥37 min)	0.704	0.653	0.357	0.703	0.652–0.755

### Univariate and Multivariate Analysis of Risk Factors for Fever

**Figure 4:**
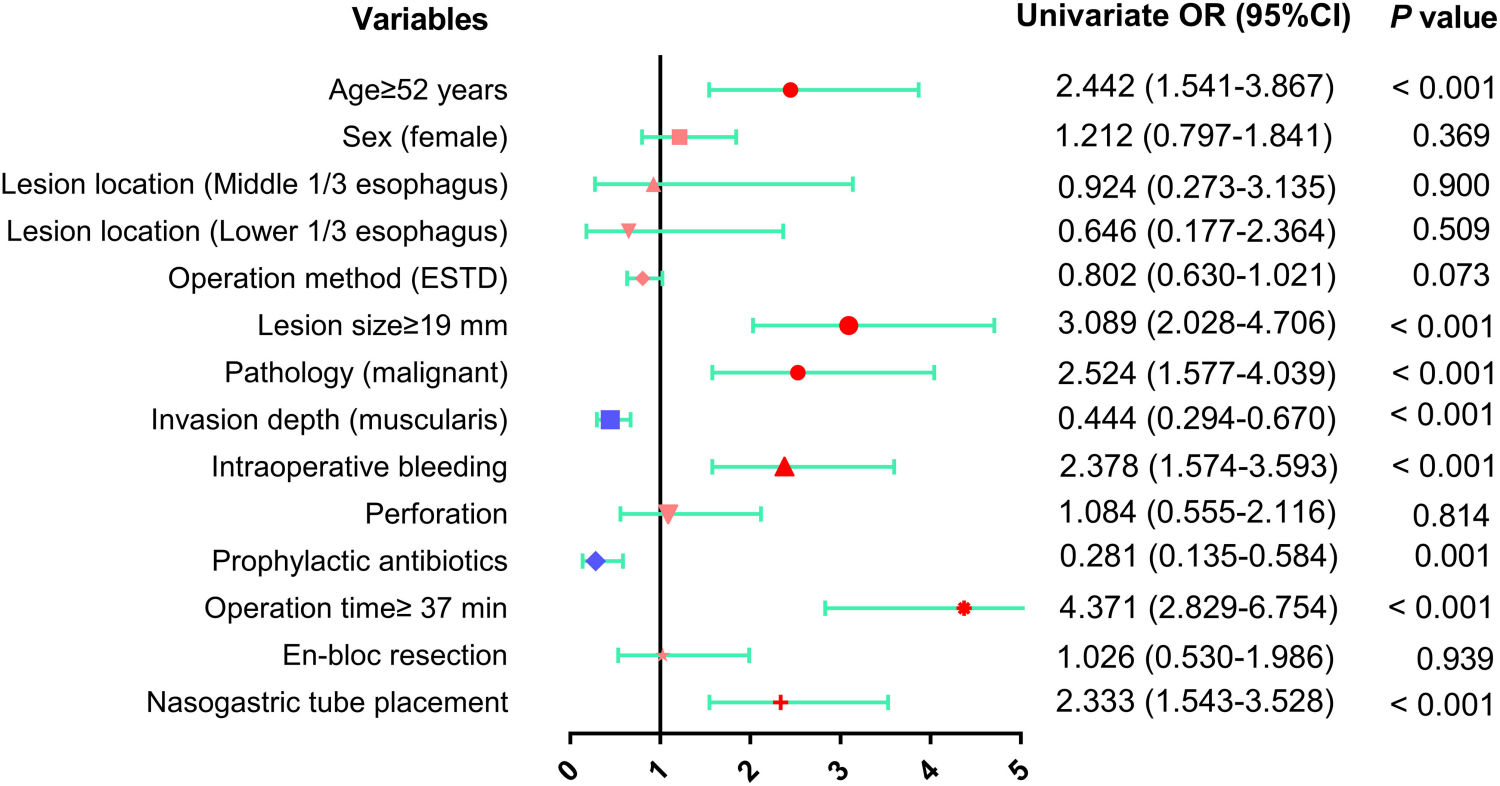
The results of univariate analysis of risk factors for fever after esophageal ESD were presented as forest plot. OR, odds ratio; CI, confidence interval.

Univariate analysis of the predictive factors for postoperative fever revealed that age ≥52 years (OR: 2.442, 95% CI: 1.541–3.867, *p* < 0.001), lesion size ≥19 mm (OR: 3.089, 95% CI: 2.028–4.706, *p* < 0.001), intraoperative bleeding (OR: 2.378, 95% CI: 1.574–3.593, *p* < 0.001), operative time ≥37 min (OR: 4.371, 95% CI: 2.829–6.754, *p* < 0.001), malignant pathology (OR: 2.524, 95% CI: 1.577–4.039, *p* < 0.001) and nasogastric tube placement (OR: 2.333, 95% CI: 1.543–3.528, *p* < 0.001) were positively correlated with the occurrence of postoperative fever, whereas invasion of the muscularis (OR: 0.379, 95% CI: 0.250–0.574, *p* < 0.001) and prophylactic antibiotic use (OR: 0.281, 95% CI: 0.135–0.584, *p* = 0.001) were negatively associated with the occurrence of fever (Table 4 and Figure 4).

**Table 4:** Univariate analysis of risk factors for fever.

**Variable**	**B**	**OR**	**95% CI**	**P-Value**
Age ≥52 (years)	0.893	2.442	1.541–3.867	<0.001
Sex (female)	0.192	1.212	0.797–1.841	0.369
**Lesion location within esophagus**				
Upper 1/3	–	–	–	ref
Middle 1/3	−0.079	0.924	0.273–3.135	0.900
Lower 1/3	−0.437	0.646	0.177–2.364	0.509
Operation method (ESTD)	−0.221	0.802	0.630–1.021	0.073
Lesion size ≥19 (mm)	1.128	3.089	2.028–4.706	<0.001
Pathology (malignant)	0.926	2.524	1.577–4.039	<0.001
Invasion depth (muscularis)	−0.812	0.444	0.294–0.670	<0.001
Intraoperative bleeding	0.866	2.378	1.574–3.593	<0.001
Perforation	0.080	1.084	0.555–2.116	0.814
Prophylactic antibiotics	−1.269	0.281	0.135–0.584	0.001
Operation time ≥37 (min)	1.475	4.371	2.829–6.754	<0.001
En-bloc resection	0.026	1.026	0.530–1.986	0.939
Nasogastric tube placement	0.847	2.333	1.543–3.528	<0.001

**Figure 5:**
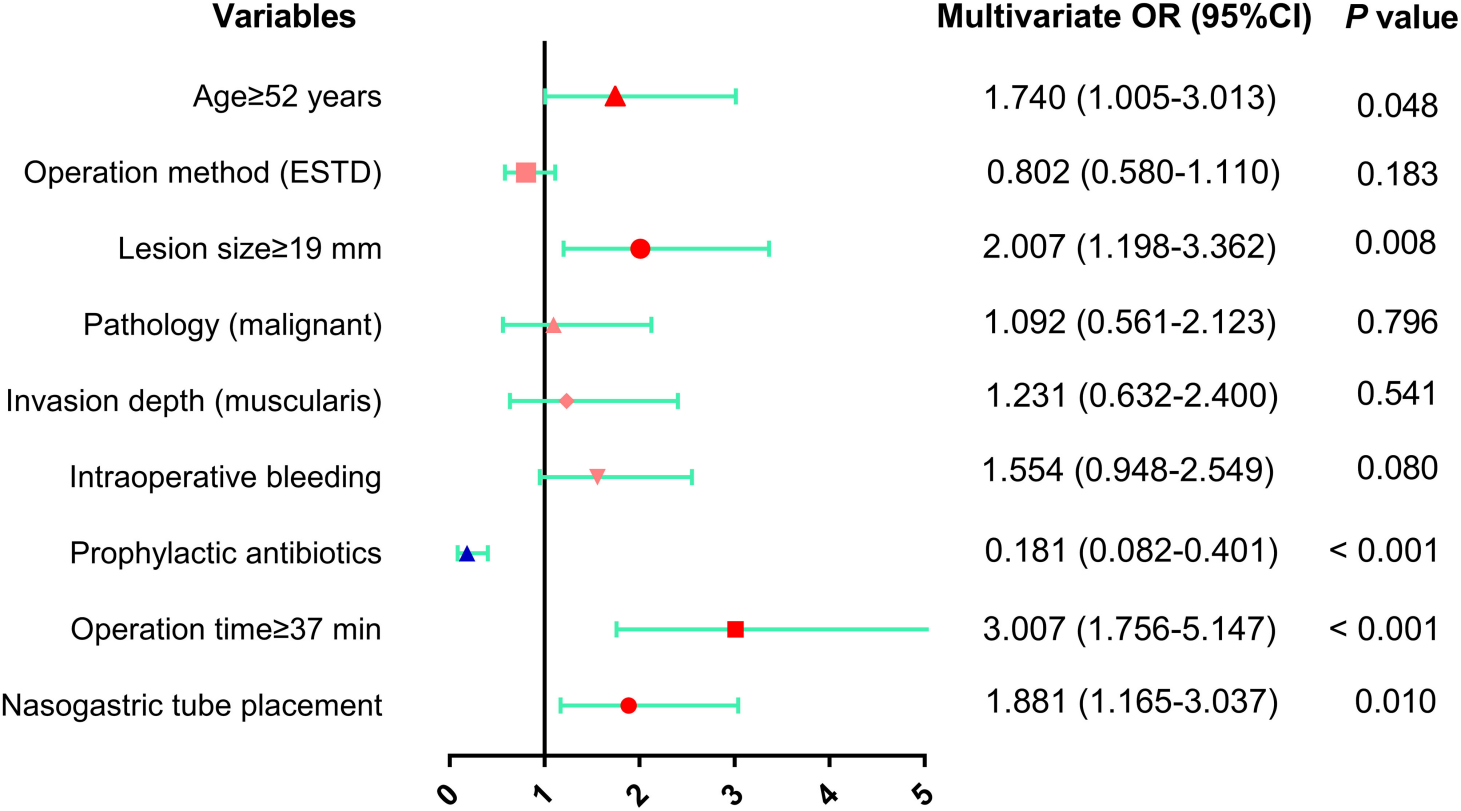
The results of multivariate analysis of risk factors for fever after esophageal ESD were presented as forest plot. OR, odds ratio; CI, confidence interval.

Predictive factors for fever onset with *p* ≤ 0.20 in the univariate analysis were subsequently examined by multivariate analysis (Table 5 and Figure 5). The results showed that 4 conditions, age ≥52 years (OR: 1.740, 95% CI: 1.005–3.013, *p* = 0.048), lesion size ≥19 mm (OR: 2.007, 95% CI: 1.198–3.362, *p* = 0.008), operation time ≥37 min (OR: 3.007, 95% CI: 1.756–5.147, *p* < 0.001) and nasogastric tube placement (OR: 1.881, 95% CI: 1.165–3.037, *p* = 0.010) were independent risk factors for fever following esophageal ESD. In contrast, there was a significant negative correlation between prophylactic antibiotic use (OR: 0.181, 95% CI: 0.082–0.401, *p* < 0.001) and fever incidence.

**Table 5:** Multivariate analysis of risk factors for fever.

**Variable**	**B**	**OR**	**95% CI**	**P-Value**
Age ≥ 52 (years)	0.554	1.740	1.005–3.013	0.048
Operation method (ESTD)	−0.222	0.802	0.580–1.110	0.183
Lesion size ≥ 19 (mm)	0.696	2.007	1.198–3.362	0.008
Pathology (malignant)	0.088	1.092	0.561–2.123	0.796
Invasion depth (muscularis)	0.208	1.231	0.632–2.400	0.541
Intraoperative bleeding	0.441	1.554	0.948–2.549	0.080
Prophylactic antibiotics	−1.707	0.181	0.082–0.401	<0.001
Operation time ≥ 37 (min)	1.101	3.007	1.756–5.147	<0.001
Nasogastric tube placement	0.632	1.881	1.165–3.037	0.010

## Discussion

Esophageal lesions include leiomyoma, squamous carcinoma, adenocarcinoma, dysplasia, stromal tumor, etc. With the development of endoscopic techniques, endoscopic resection has become a standard treatment method for these gastrointestinal tumors (22, 23). Compared to endoscopic mucosal resection (EMR), ESD results in increased en-bloc and curative resection rates for the treatment of esophageal lesions, but adverse events (such as perforation) are more likely to occur after ESD than EMR (24), which may be related to the complexity of ESD operative procedures (25). After ESD, we often focus on serious adverse events, such as bleeding and perforation, while postoperative fever is also common but has not been fully assessed. Therefore, we designed this study to investigate the postoperative fever rate and risk factors in patients with esophageal lesions.

The incidence of postoperative fever in this study was 30.3% (135/446), higher than reported in previous studies, which varied from 10 to 24.8% (12, 26). There are several potential reasons for the high fever rate in our study, one of which is that our definition of fever differs from that of a previous study (fever as body temperature ≥38°C) (26). It may also be related to the anatomy of the esophagus itself, which lacks a protective serous layer. Clinically, postoperative fever is often considered to be related to infection, which may be caused by ESD-related endotoxemia (27). Patients under anesthesia and sedation during ESD may also induce aspiration pneumonia and lead to fever (28). In addition, wound tissue damage may also be one of the causes of fever. Among patients with fever, they have higher likelihood of bleeding, that was likely treated with electrocautery. Intraoperative bleeding may require more thermal coagulation, causing inflammatory responses and leading to fever. Our data also suggest that not all fevers are indicative of infection, and that more than half of patients can return to normal body temperature through clinical observation and physical cooling. Although 135 patients developed fever after ESD/ESTD, there is no serious complications related to fever occurred through our timely treatment. All patients returned to normal body temperature after our treatment. Usually, when the fever is <38.5°C, we will take the way of clinical observation or physical cooling. Laboratory tests such as blood routine, CRP, procalcitonin, and blood culture should be performed immediately when the patient's body temperature ≥38.5°C or conventional management measures fail. Infection is considered present when a patient's body temperature is higher than 38.5°C, combined with procalcitonin ≥0.5 ng/ml or a positive blood culture, and antibiotics are used to control infection. In the present study, we found that age ≥52 years, lesion size ≥19 mm, operation time ≥37 min and nasogastric tube placement were risk factors for postoperative fever, while prophylactic antibiotic administration was negatively associated with fever. Nakanishi et al. (12) included 471 ESD patients with gastric lesions, demonstrating that age and resection diameter were risk factors for pyrexia in patients without pneumonia, and operation time was a risk factor for pyrexia in patients with pneumonia. A previous study involving 199 patients undergoing ESD due to colon lesions found that age and lesion size were closely associated with postoperative fever, but the possibility of bacteremia causing fever is very low (13). These data support our results. We believe that susceptibility of the elderly to fever may be related to reduced immunity and the presence of more underlying diseases. Furthermore, large mucosal lesions are likely to cause fever, because they often lead to increased mucosal damage and require more thermal energy. Takeuchi et al. (29) assert that large tumor size is closely related to ESD operation time because it is often accompanied by increased surgical difficulty and leads to greater mucosal defects. This also seems to explain that ESD operation time ≥37 min was a risk factor for postoperative fever in our study. In addition, in the present study, we found that nasogastric tube placement was another significant independent risk factor for pyrexia. Although no previous studies have suggested that nasogastric tube placement is associated with fever, prolonged placement of a nasogastric tube may lead to iatrogenic infection. More research may be needed to investigate this. We observed a negative correlation between prophylactic antibiotics and postoperative fever. A previous article (26) indicated that the incidence of bacteremia after esophageal ESD was low, so there is no need for routine prophylactic antibiotics for patients undergoing ESD. However, in actual clinical work, we inevitably use prophylactic antibiotics in some patients with a high risk for fever. Previous studies have never included prophylactic antibiotic use in risk factor analysis. We included and analyzed prophylactic antibiotic administration in the study and concluded that prophylactic antibiotics were negative correlated with fever incidence. This also suggests that prophylactic antibiotics can be used in clinical practice in patients at high risk of fever to reduce the incidence of fever after operation. We think the intramuscular layer of ESD surgery tends to cause more complications (30), but an interesting phenomenon emerged in our study. In the univariate analysis, lesions confined to the mucosa/submucosa layer were significantly associated with postoperative fever, in contrast to several studies (30, 31). However, when we incorporated variables, such as operation method, into the multivariate analysis, lesion localization to the mucosa/submucosa was not an independent risk factor for fever. A meta-analysis by Zhang et al. (32) suggested that compared to ESD, ESTD significantly reduces postoperative adverse events. Similarly, in the current study, a tendency toward higher ESD operation rate was observed in the fever group. Therefore, such bias was eliminated when we included the operation method in the multivariate analysis.

The advantages of the current study include systematic analysis of the incidence and risk factors for fever after esophageal ESD/ESTD. However, the present study does have several limitations. First, this analysis was a single-center retrospective study. Therefore, a multicenter prospective study should be conducted. Second, because this is a retrospective study, the data we registered may have errors compared to the actual situation, affecting the accuracy of the study. Third, the number of patients included in this study is relatively small, so a study with large sample size is still needed.

## Conclusions

In conclusion, age ≥52 years, lesion size ≥19 mm, operation time ≥37 min and nasogastric tube placement were independent risk factors of postoperative fever, prophylactic antibiotic use after operation may help reduce fever rate. Given these findings, we should pay more attention to patients who have these factors.

## Data Availability Statement

The raw data supporting the conclusions of this article will be made available by the authors, without undue reservation.

## Ethics Statement

The studies involving human participants were reviewed and approved by the Ethics Committee of the First Affiliated Hospital of Nanchang University. Written informed consent to participate in this study was provided by the participants' legal guardian/next of kin.

## Author Contributions

FL collected data, analyzed relevant information, and drafted the manuscript. ZZ designed the study and collected the data. YL collected the data. XP, SL, XZ, GL, YZ, and YC clinically managed the patient. XS designed the article, approved the final submission, and clinically managed the patient. All authors contributed to the article and approved the submitted version.

## Conflict of Interest

The authors declare that the research was conducted in the absence of any commercial or financial relationships that could be construed as a potential conflict of interest.

## Publisher's Note

All claims expressed in this article are solely those of the authors and do not necessarily represent those of their affiliated organizations, or those of the publisher, the editors and the reviewers. Any product that may be evaluated in this article, or claim that may be made by its manufacturer, is not guaranteed or endorsed by the publisher.

## Supplementary Material

Supplementary Figure 1 Receiver operating characteristic (ROC) curves were plotted to determine the optimal cut-off values for age, lesion size and operation time to predict fever.
