# Supplementary figures and images for: Risk Factors for Fever After Esophageal Endoscopic Submucosal Dissection and Its Derived Technique
Source: Front Med (Lausanne). 2022 Feb 22;9:713211. doi: 10.3389/fmed.2022.713211 (PMC8902360; doi:10.3389/fmed.2022.713211)

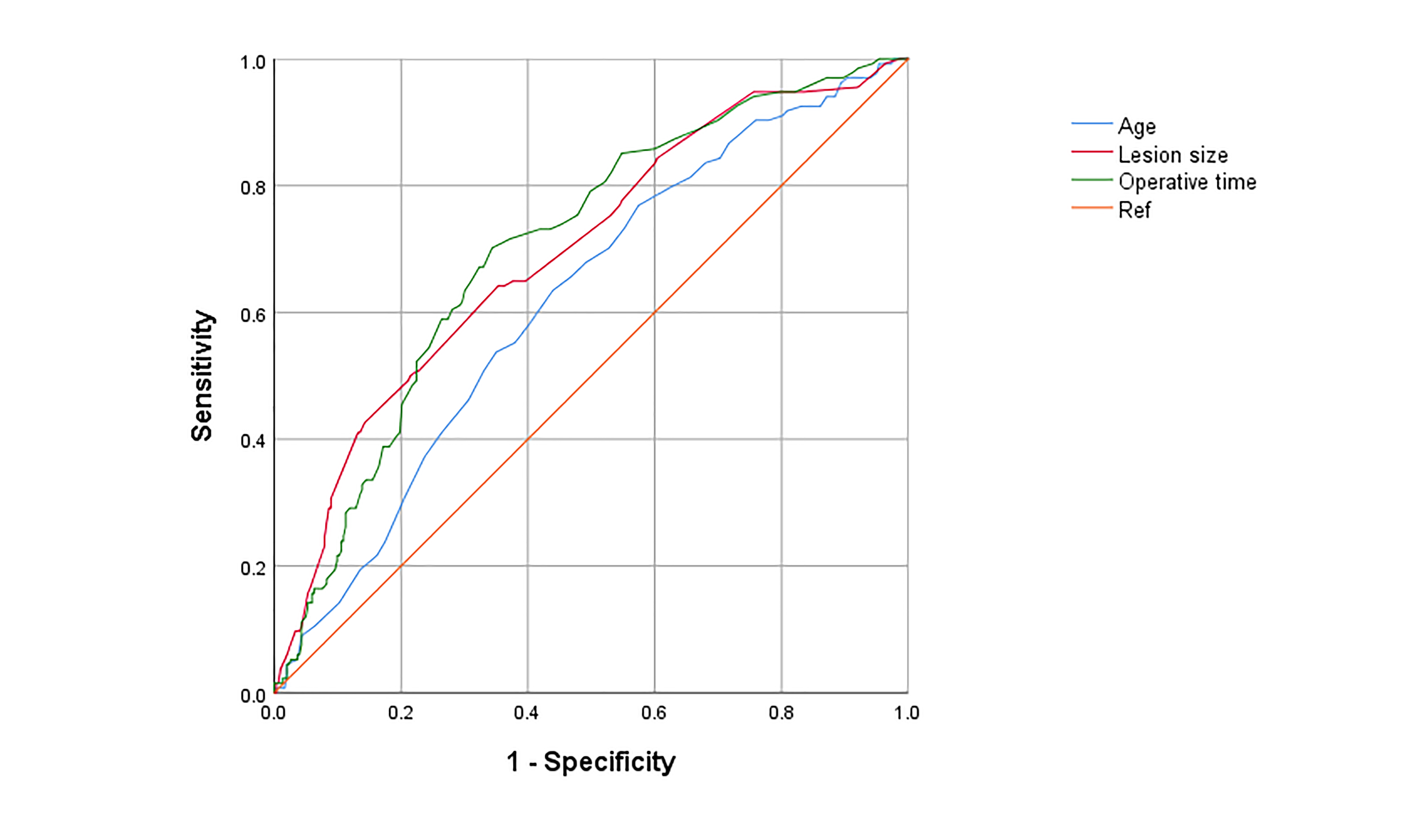

Supplement: Supplementary Figure 1 — Receiver operating characteristic (ROC) curves were plotted to determine the optimal cut-off values for age, lesion size and operation time to predict fever. [file Image_1.TIF]
