# Supplementary material for: Risk Factors for Fever After Esophageal Endoscopic Submucosal Dissection and Its Derived Technique
Source: Front Med (Lausanne). 2022 Feb 22;9:713211. doi: 10.3389/fmed.2022.713211 (PMC8902360; doi:10.3389/fmed.2022.713211)
Supplement: Supplementary file 2 [file Data_Sheet_1.docx]

Contribution to the Field Statement

Fever is a common postoperative complications of endoscopic submucosal dissection. Due to the special anatomical structure of the esophagus and its lack of a protective serous layer, the possibility of fever after esophageal endoscopic submucosal dissection is greater than that in other parts of the digestive tract. At present, there were few studies on risk factors for fever after esophageal endoscopic submucosal dissection and its derived technique. Our study found that age, lesion size, operation time and gastric catheterization are risk factors for postoperative fever, and prophylactic antibiotic use after operation may help reduce fever rate**.** This can help us optimize preoperative risk assessment and enhance intraoperative and postoperative management, so as to reduce postoperative fever rate, improve patient prognosis, and reduce hospital stay and cost.
